# Towards quantitative intensity analysis of conventional T1-weighted images in multiple sclerosis

**DOI:** 10.1016/j.neuroimage.2025.121395

**Published:** 2025-07-23

**Authors:** Tun Wiltgen, Julian McGinnis, Ronja Berg, Cui Ci Voon, Oula Puonti, Katrin Giglhuber, Carl Ganter, Claus Zimmer, Bernhard Hemmer, Benedikt Wiestler, Jan Kirschke, Christine Preibisch, Mark Mühlau

**Affiliations:** a School of Medicine and Health, Department of Neurology, Technical University of Munich, Munich, Germany; b School of Medicine and Health, TUM-Neuroimaging Center, Technical University of Munich, Munich, Germany; c Chair for AI in Healthcare and Medicine, TUM University Hospital, Technical University of Munich, Munich, Germany; d School of Medicine and Health, Institute for Diagnostic and Interventional Neuroradiology, Technical University of Munich, Munich, Germany; e Danish Research Centre for Magnetic Resonance, Center for Functional and Diagnostic Imaging and Research, Copenhagen University Hospital Hvidovre, Denmark; f Athinoula A. Martinos Center for Biomedical Imaging, Massachusetts General Hospital, Boston, MA, USA; g School of Medicine and Health, Department of Diagnostic and Interventional Radiology, Technical University of Munich, Munich, Germany; h Munich Cluster for Systems Neurology (SyNergy), Munich, Germany; i School of Medicine and Health, AI for Image-Guided Diagnosis and Therapy, Technical University of Munich, Munich, Germany; j Munich Center for Machine Learning (MCML), Munich, Germany

**Keywords:** T1-weighted MRI, Multiple sclerosis, Intensity scaling, Quantitative MRI, Normal-appearing white matter

## Abstract

Conventional T1-weighted (T1w) magnetic resonance imaging (MRI) is commonly used in multiple sclerosis (MS) morphometry and volumetry research. However, arbitrary intensity scales preclude interpretation of signal values across patients, sites, and time. This requires quantitative MRI techniques, which are not always available.

This study assessed T1w image intensity scaling methods, relying on extracerebral reference regions, for quantitative analysis of brain MRI in MS.

In total, 701 people with a diagnosis of radiologically isolated syndrome, clinically isolated syndrome, or MS were included. Four intensity scaling strategies were applied: 1) MRI signal modeling, 2) linear scaling with reference regions, 3) z-score standardization, and 4) none (only bias field correction). Methods were evaluated using variance analysis, R1 map comparison, and normal-appearing white matter (NAWM) intensity group comparison, using mean and coefficient of variation (CoV), between low (≤3) and high (>3) expanded disability status scale (EDSS) scores. Statistical analysis was conducted using Pearson’s r, two-sided Welch two-sample *t*-test, ANCOVA, and Cohen’s d.

Linear scaling with temporal fatty tissue achieved the most consistent variance reduction and strong correlation with R1 maps (*r* = 0.84). R1 values in NAWM were significantly lower in people with high compared to low EDSS scores (*d* = −0.351). Similarly, group differences in mean NAWM intensity of fat-scaled images were significant (*d* = −0.252). The largest group differences were found in NAWM CoV in bias field-corrected T1w images (*d* = 0.818).

Linear scaling with fatty tissue most accurately reproduced the results obtained with R1 maps. Changes in MS NAWM appear to increase intensity variability detectable in conventional T1w images.

## 1. Introduction

In clinical practice, magnetic resonance imaging (MRI) is one of the main tools to diagnose and monitor neurological diseases such as multiple sclerosis (MS) (Thompson et al., 2018). Conventional MRI is mainly used to detect abnormalities such as demyelinating white matter (WM) lesions that appear hyperintense in T2-weighted (T2w) images and vary between hypo- and isointense in T1-weighted (T1w) images. However, disease-related tissue damage (not only in MS) often extends beyond what can be detected by the human eye on conventional MRI. In MS, histological studies substantiated pathological processes in WM regions that appear normal in conventional MRI (Kutzelnigg et al., 2005; Laule et al., 2013; Moll et al., 2011). Analyzing normal-appearing white matter (NAWM) with respect to tissue damage requires image intensities on an absolute quantitative scale, making them interpretable and comparable between images and subjects. However, conventional T1w MRI is optimized for good visual contrast, with arbitrary image intensity scales influenced by multiple factors, such as hardware-specific settings (e.g., coils, manufacturer, signal amplifier gain), scanner software, and patient anatomy and positioning. Therefore, quantitative MRI sequences have been developed using mathematical models and calibration techniques. Numerous studies have shown that advanced quantitative MRI, such as R1 relaxometry, is sensitive to tissue damage in MS NAWM (Ladopoulos et al., 2022; Lommers et al., 2019; MacKay et al., 2009; Manfredonia et al., 2007; O’Muircheartaigh et al., 2019; Pontillo et al., 2023; Vrenken et al., 2006). Yet, acquiring quantitative MRI is not widely integrated into clinical routines because acquisition is time-consuming and off-line evaluation is frequently required. Given these limitations and that T1w data is widely available, recent studies have attempted to derive quantitative images from conventional T1w MRI using various intensity scaling approaches (Brown et al., 2020; Fortin et al., 2016; Ganzetti et al., 2014; Ghassemi et al., 2015; Hasse et al., 2022; Lavielle et al., 2024, 2023; Lee et al., 2015; Mejia et al., 2016; Moskovich et al., 2024; Shinohara et al., 2014; Wahid et al., 2021). Ideally, intensity scaling removes technically induced inter-image variance while preserving the biologically induced variance that contains information on the disease under investigation. Some approaches are based on reference regions that ideally should only reflect technical variance, which could then be modeled and compensated for independently of the biological variance. Most frequently, intensities or scaling factors are derived from reference regions within the brain, which can be critical in studies focusing on brain diseases. In the case of MS, regions outside of the brain are less likely to be directly affected by the disease and are, hence, attractive candidates for reference regions. In our recently published systematic review (Wiltgen et al., 2024b), we discussed studies relying on intensity scaling with extracerebral reference regions such as various fatty tissues, temporal muscle, and eyes (Brown et al., 2020; Ganzetti et al., 2014; Ghassemi et al., 2015; Gilmore et al., 2007; Lee et al., 2015) and found that these have shown promising results.

However, validation studies comparing different approaches or relating images with scaled intensities to quantitative MRI are sparse. Some studies aimed to validate intensity scaling methods through comparison with (semi-) quantitative MRI, such as the magnetization transfer ratio or diffusion-weighted imaging (Brown et al., 2020; Ganzetti et al., 2014; Gilmore et al., 2007). Other studies modeled the T1-dependency of T1w MRI signals to estimate T1 properties and validated them against actual T1 or R1 maps (Hasse et al., 2022; Lavielle et al., 2024, 2023; Mejia et al., 2016).

In this study, we aimed to compare and validate several approaches for intensity scaling of conventional T1w images: 1) MRI signal modeling, 2) linear scaling with extracerebral reference regions, 3) z-score standardization, and 4) none (only bias field correction). Technical validation assessed the reduction of inter-image variance and the correlation between scaled T1w images and R1 maps. Biological validation assessed each method’s ability to detect differences in mean NAWM intensity between people with MS with low (≤3) and high scores (>3) on the expanded disability status scale (EDSS). Finally, we contrasted the group comparisons of mean intensities, a robust and global measure, with the intensity variability across NAWM using the coefficient of variation (CoV). This approach was inspired by the observation of Johnson et al. (Johnson et al., 2023) that the CoV of myelin water fraction (MWF) in NAWM better distinguishes people with MS from healthy controls than mean values. Building on this, and since the CoV is independent of the absolute intensity scale, we hypothesized that greater intensity variability in NAWM of conventional T1w images may already reflect a decreased tissue integrity.

## 2. Methods

**Fig. 1.**
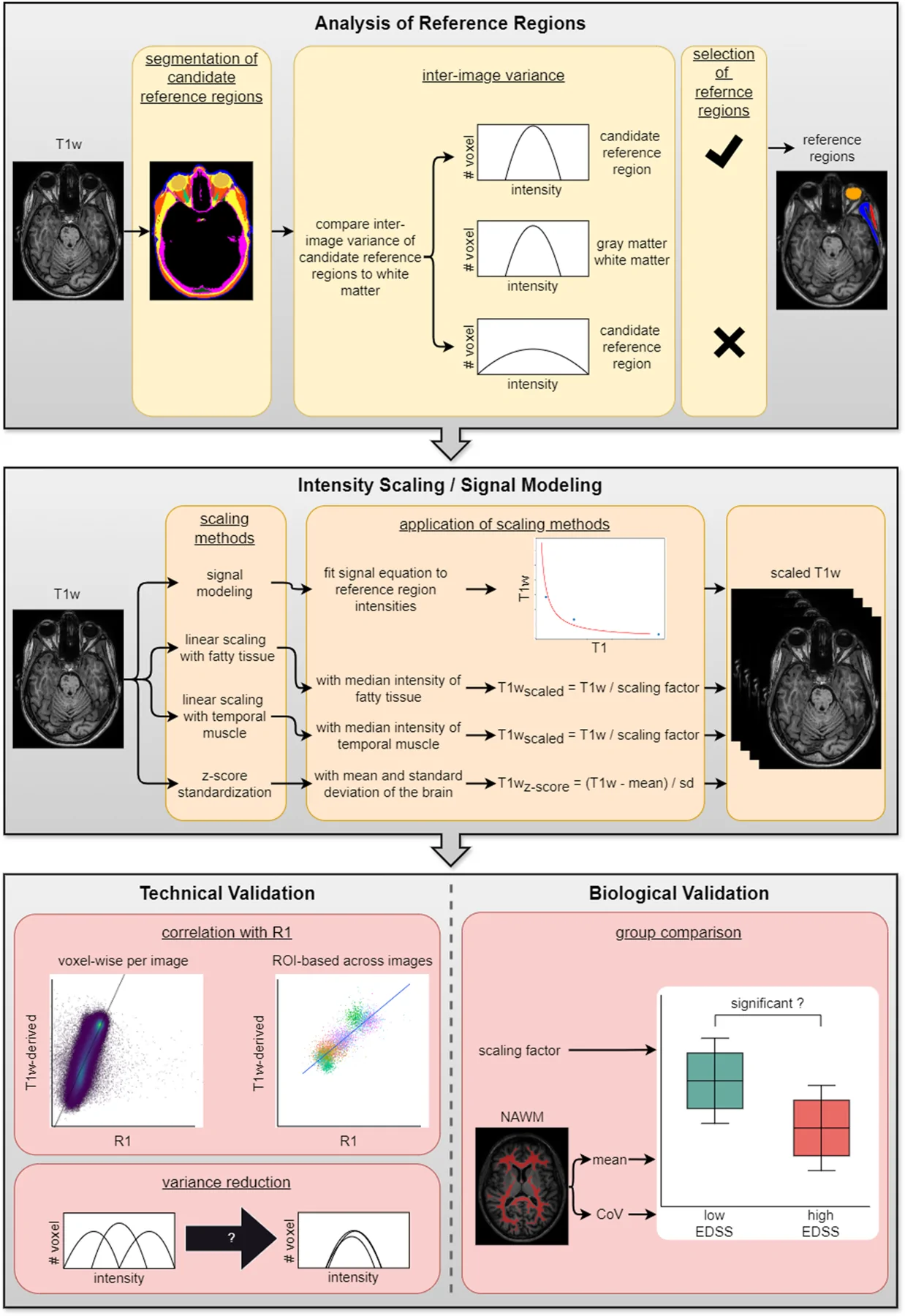
Workflow overview. Overview of the workflow. First (top panel), potential extracerebral reference regions were selected with a data-driven approach. Multiple candidate reference regions were segmented, and inter-image variance was quantified with the CoV and compared with GM and WM. Only regions with equal or lower CoVs were selected. Second (middle panel), different scaling methods were applied to bias-corrected T1w images. These methods included MRI signal modeling, linear scaling with a reference region, and z-score standardization. Third (bottom panel), scaling methods were evaluated through technical and biological validation. For technical validation (left), scaled T1w images were compared to quantitative R1 maps, and the inter-image variance was analyzed. Biological validation (right) comprised group comparisons of NAWM intensities (with mean and CoV) between subjects with low (≤3) and high (>3) EDSS scores. In addition, scaling factors were tested for group differences to confirm that scaling methods are not influenced by disease severity. Abbreviations: CoV: coefficient of variation, EDSS: expanded disability status scale, GM: gray matter, MRI: magnetic resonance imaging, NAWM: normal-appearing white matter, ROI: region of interest, sd: standard deviation, T1w: T1-weighted, WM: white matter.

This study was conducted in accordance with the Code of Ethics of the World Medical Association (Declaration of Helsinki) for experiments involving humans (World Medical Association, 2001). Patients gave written informed consent to use their data for research purposes. An overview of the study workflow is presented in Fig. 1.

### 2.1. Data sets

In the total cohort, 1078 image data sets of 701 subjects with confirmed radiologically isolated syndrome (RIS), clinically isolated syndrome (CIS), or MS diagnosis according to the McDonald criteria (Thompson et al., 2018) from our in-house database were included. Further inclusion criteria were MRI acquisition on the same scanner and 18 years or older. The total cohort was used to identify valid reference regions.

A subset of 597 image data sets of 597 subjects (validation cohort) was used for the technical and biological validation of intensity scaling methods. It only included people with CIS or MS whose EDSS scores were acquired within 31 days before or after the MRI scan. When multiple image sessions were available for one subject, we selected the most recent data to increase sensitivity to disease-related effects. In the context of biological validation, the validation cohort was divided into two groups: low (≤3) and high (>3) EDSS (Leray et al., 2010). Details of the cohorts are presented in Table 1.

### 2.2. MRI acquisition

All subjects underwent conventional MRI and multiparameter mapping (MPM) (Berg et al., 2022; Leutritz et al., 2020; Tabelow et al., 2019), acquired on a Philips 3.0 T Ingenia scanner (Philips Medical Systems, Best, The Netherlands) within a time window of 21 months, during which no hard- or software upgrades were applied. Conventional imaging included a 3D T1w magnetization-prepared rapid gradient echo (MPRAGE) sequence and a 3D T2w fluid-attenuated inversion recovery (FLAIR) sequence. MPM data were acquired with a 3D radiofrequency-spoiled multi-echo gradient-echo sequence (fast field echo) with six echoes, using a Compressed SENSE acceleration factor of 6 (Berg et al., 2022; Geerts-Ossevoort et al., 2018). Within each MPM acquisition, four protocols were included: 1) B1 mapping for voxel-wise bias field correction, 2) T1w, 3) proton density-weighted (PDw), and 4) magnetization transfer-weighted (MTw). Further technical details have been described in a previous study (Berg et al., 2022). MRI parameters are presented in Table 2.

### 2.3. Image processing

First, N4 bias field correction (Tustison et al., 2010), implemented in the Advanced Normalization Tools (ANTs) software (https://github.com/ANTsX/ANTs), was applied to all T1w images. Sequence Adaptive Multimodal SEGmentation (SAMSEG) software integrated into Freesurfer version 7.3.2 (Cerri et al., 2021; Fischl, 2012; Puonti et al., 2016) was used to segment WM, cortical gray matter (GM), cerebellum GM and WM, accumbens, amygdala, caudate, hippocampus, putamen, and thalamus. The inputs were pairs of registered T1w and FLAIR images, co-registered using “mri_coreg” and “mri_vol2vol” integrated into Freesurfer. NAWM masks were created by first applying WM lesion segmentation with LST-AI (Wiltgen et al., 2024a) using non-registered T1w and FLAIR images as input, as registration is integrated into LST-AI. Next, the lesion mask was subtracted from the SAMSEG WM segmentation. The mask was further eroded using a 3 × 3 × 3 cube as a structuring element, resulting in a conservative segmentation of NAWM. In addition, whole-brain binary masks were generated with the HD-BET brain extraction tool (Isensee et al., 2019) using T1w images as input.

Candidate reference regions were segmented using SAMSEG combined with an atlas comprising extracerebral tissues, similar to the approach by Puonti et al. (Puonti et al., 2020). Details on the atlas generation are provided in the supplementary material. The extracerebral tissues included cancellous bone, cortical bone, eyes, optic nerves, rectus muscles, and skin. In addition, temporal fatty tissue and temporal muscle were segmented as they are distinctly visible in conventional T1w MRI, and previous studies have also used fatty tissue and temporal muscle for scaling (Brown et al., 2020; Ganzetti et al., 2014; Ghassemi et al., 2015; Gilmore et al., 2007; Lee et al., 2015). For precise and efficient segmentation of fatty tissue and temporal muscle, we trained two 3D nnUNets (Isensee et al., 2021) with a training data set of 166 manually segmented masks (see supplementary material), conducted with 3D Slicer version 5.6.2 (Fedorov et al., 2012, https://www.slicer.org/). Masks were generated for the total cohort through inference using the trained models and manually corrected if necessary. In general, all images and segmentation masks underwent a thorough visual quality check.

Longitudinal relaxation rate (R1 = 1/T1) maps were computed with the hMRI (Tabelow et al., 2019) toolbox (version: v0.1.3-dev; RRID: SCR_017682; https://github.com/tleutritz-cbs/hMRI-toolbox) implemented in the Statistical Parametric Mapping (SPM12) framework (https://www.fil.ion.ucl.ac.uk/spm/software/spm12/), using the same workflow as in (Berg et al., 2022). The quantitative R1 maps were rigidly registered to the Montreal Neurological Institute (MNI) space using the “Auto-Reorient” application included in the hMRI toolbox.

T1w images were rigidly registered to R1 maps in MNI space, using the Greedy command line tool (Yushkevich et al., 2016, 2006; https://github.com/pyushkevich/greedy), allowing for comparison of intensity-scaled T1w images with R1 maps. The resulting transformation matrices were used to register the intensity-scaled T1w images and segmentations to the respective R1 maps.

### 2.4. Scaling of conventional MRI

#### 2.4.1. MRI signal modeling

This method builds on a physical model of MRI signal generation, similar to approaches in previous studies (Hasse et al., 2022; Lavielle et al., 2023; Moskovich et al., 2024). The signal equation relates the acquired MRI signal to physical properties, such as T1 relaxation times (see detailed derivation and sequence diagram in supplementary material). For an MPRAGE sequence with echo time TE, flip angle *α*, repetition time TR, recovery time T_rec_, and inversion time TI, the signal after the i^th^ inversion pulse – obtained from a sample with longitudinal and effective transverse relaxation time T1 and T2*, respectively – is given by (Brant-Zawadzki et al., 1992; Deichmann et al., 2000; Deichmann and Haase, 1992; Haase, 1990; Vernier et al., 2021):

(1) $$S_{i}=\text{sin}(\alpha )*M_{i}^{N}*\text{exp}\left(-\text{TE}/T2^{*}\right)$$

with $M_{i}^{N}$ being the longitudinal magnetization after the N^th^ radio frequency pulse in the rapid gradient echo block:

(2) $$M_{i}^{N}=M_{0}\left(1-e^{-\frac{TR}{T1}}\right)*\frac{1-{\left(\text{cos}(\alpha )*e^{-\frac{TR}{T1}}\right)}^{N}}{1-\text{cos}(\alpha )*e^{-\frac{TR}{T1}}}\;+\left(M_{0}\left(1-e^{-\frac{TI}{T1}}\right)-M^{eq}*e^{-\frac{TI}{T1}}\right)*{\left(\text{cos}(\alpha )*e^{-\frac{TR}{T1}}\right)}^{N}$$

with the steady state equilibrium magnetization *M*^*eq*^:

(3) $$M^{eq}=M_{0}\left(*\frac{1-e^{-\frac{\text{Trec}}{T1}}+\left(1-e^{-\frac{TR}{T1}}\right)*\frac{1-{\left(\text{cos}(\alpha )*e^{-\frac{TR}{T1}}\right)}^{N_{\text{total}}}}{1-\text{cos}\left(\alpha \right)*e^{-\frac{TR}{T1}}}*e^{-\frac{\text{Trec}}{T1}}}{1+{\left(\text{cos}\left(\alpha \right)*e^{-\frac{TR}{T1}}\right)}^{N_{\text{total}}}*e^{-\frac{TI}{T1}}*e^{-\frac{\text{Trec}}{T1}}}+\frac{\left(1-e^{-\frac{TI}{T1}}\right)*{\left(\text{cos}(\alpha )*e^{-\frac{TR}{T1}}\right)}^{N_{\text{total}}}*e^{-\frac{\text{Trec}}{T1}}}{1+{\left(\text{cos}\left(\alpha \right)*e^{-\frac{TR}{T1}}\right)}^{N_{\text{total}}}*e^{-\frac{TI}{T1}}*e^{-\frac{\text{Trec}}{T1}}}\right)$$

*M*_0_ represents the acquisition-specific equilibrium magnetization, which depends on the spin density (*ρ*) and on technical properties (*k*) such as receiver amplifier gain and coil design. This can be expressed as: *M*_0_ = *k* *ρ. For simplification, we relied on the MPRAGE sequence’s characteristics, which leverage strong T1 contrast and minimize the impact of *ρ* differences across tissues, allowing for the approximation of *ρ* ≈ *constant* without compromising contrast accuracy (Haase, 1990). Consequently, we consider *M*_0_ as uniform across the entire image.

Based on equations [1–3], the theoretically expected T1w signal could be calculated if the equilibrium magnetization *M*_0_, relaxation times (T1, T2*), and imaging parameters (TE, *α*, TR, T_rec_, and TI) were known. We neglected the influence of T2* relaxation and flip angle inhomogeneity because TE is sufficiently short (TE = 4 ms) and we applied bias field correction of T1w images. Since equation [1] is a formally non-invertible function, *M*_0_ was numerically estimated using pairs of T1 and T1w values. First, for each reference region, a fixed T1 value was determined by extracting the median T1 value from that region in each individual R1 map (T1 = 1/R1) and then computing the median of these values across all data sets (*n* = 1078). Second, median T1w intensities were extracted from reference regions for each bias-corrected T1w image. The signal equation was fitted to these data pairs using orthogonal distance regression, estimating each image’s *M*_0_ constant. The image-specific *M*_0_ value was then used to calculate the 1-to-1 mapping between T1w intensities and T1 values. This was realized by sampling T1 values across a range covering all relevant tissues, from 200 ms (lower than fatty tissue) to 4500 ms (approximating cerebrospinal fluid) (Bojorquez et al., 2017), and calculating the corresponding T1w intensities. The 1-to-1 mapping was then used to generate estimated R1 maps, where each voxel was assigned the inverse T1 value (R1 = 1/T1) based on its actual T1w intensity.

#### 2.4.2. Linear scaling with reference regions

Linear scaling divides the entire image by a scaling factor, defined as the median intensity of a reference region, ideally removing technically induced inter-image variance while preserving biological variance:

$$T1w_{\text{scaled}}=T1w/\text{scaling}\;\text{factor}$$

#### 2.4.3. z-score standardization

Z-score standardization is popular in medical image processing and eliminates intensity scale discrepancies by centering the data around zero and transforming it to units of standard deviation:

$$T1w_{\text{zscore}}=\left(T1w–\text{mean}\right)/\text{standard}\;\text{deviation}$$

We calculated the mean intensity and standard deviation within the binary brain masks generated with HD-BET. The brain masks cover cerebral and cerebellar WM and GM, and CSF.

### 2.5. Analysis and validation

#### 2.5.1. Analysis of reference regions

A data-driven approach was used to identify suitable reference regions. As intensity scaling methods aim to convert image intensities into comparable, quantitative units, especially within the brain, the reference regions should not exhibit a higher inter-image variance than brain regions such as the cerebral cortex or WM. Regions with higher variance are expected to contain confounding biological variance, hampering the estimation of technical variance. The inter-image variance of candidate reference regions was measured with the CoV of median intensities across all bias-corrected T1w images (*n* = 1078) and compared to WM and cortical GM.

#### 2.5.2. Technical validation

The first step assessed the potential of intensity scaling methods to reduce inter-image variance. Therefore, for each region of interest (ROI) within the brain, the variance of median intensities was calculated for each scaling method using the CoV across images (*n* = 597).

The second step assessed the ability to convert conventional T1w image intensities into quantitative units with correlation analysis between intensity-scaled T1w images and quantitative R1 maps (*n* = 597). Two approaches were used: 1) calculating the median intensity of each brain ROI per image and computing Pearson’s r across all ROIs and images, and 2) computing voxel-wise Pearson’s r between R1 maps and intensity-scaled T1w images per data set, then averaging across Pearson’s r values.

#### 2.5.3. Biological validation

The difference of each scaling factor between low (*n* = 526) and high (*n* = 71) EDSS groups was assessed to ensure their independence from disease severity. Then, for each intensity scaling method, the mean intensity and the CoV were extracted from NAWM and compared between low and high EDSS groups. The two-sided Welch’s *t*-test was used for group comparisons. Cohen’s d was calculated to measure the effect size of the group differences (low vs. high EDSS). For the NAWM comparison, we also applied ANCOVA, including age, sex, disease duration, and total lesion volume as covariates.

## 3. Results

### 3.1. Analysis of reference regions

**Fig. 2.**
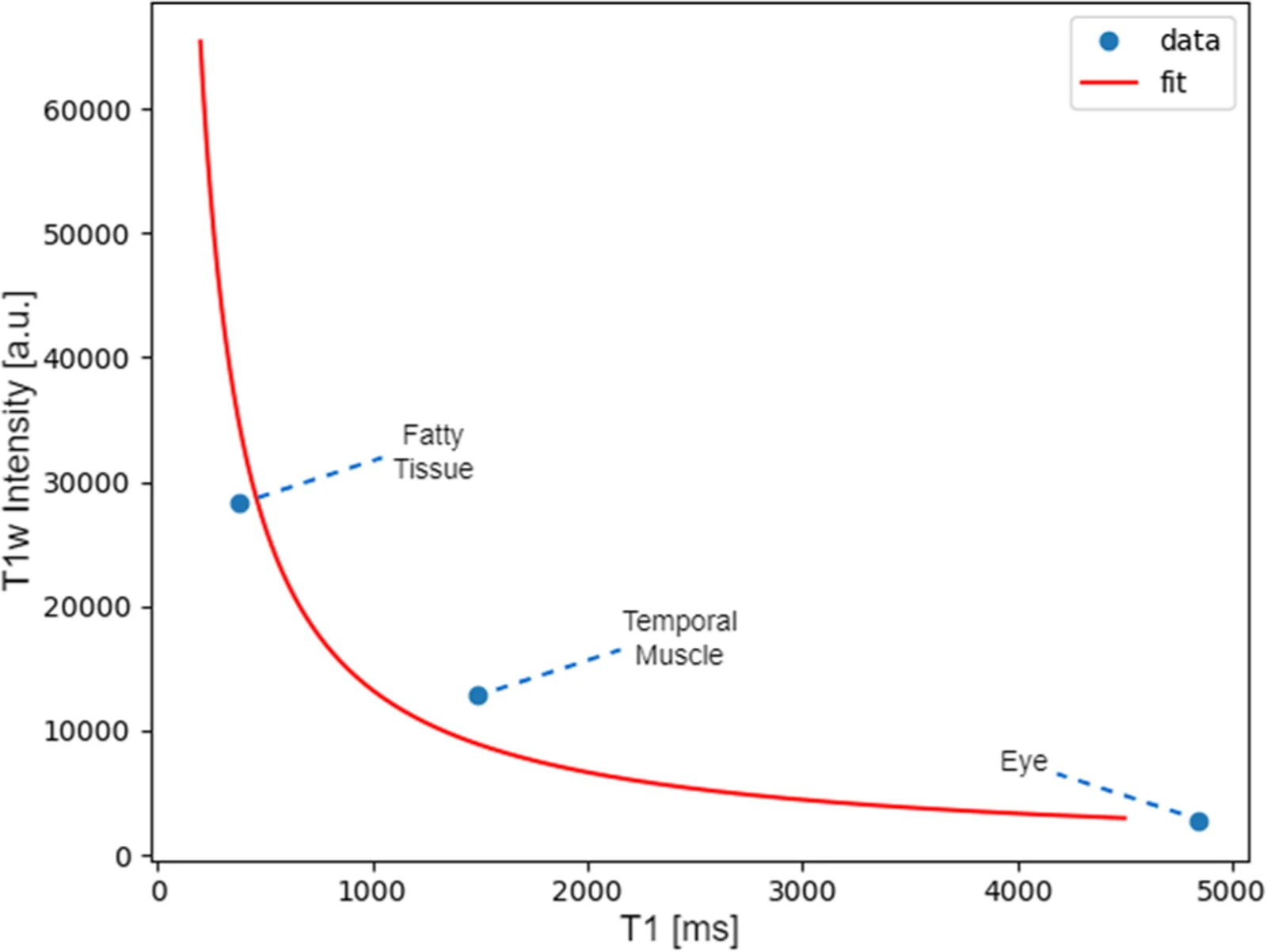
MPRAGE signal modeling. The red curve shows how the MPRAGE signal was fitted to data from reference regions (fatty tissue, temporal muscle, and eye). The blue points represent, for each reference region, data points defined by pairs of fixed median intensity extracted from T1 maps and median intensity extracted from a specific bias-corrected T1w image. Notice how each data point provides a reference in a different intensity range, sampling the MRI signal spectrum in the low-, mid-, and high-intensity range. Abbreviations: MPRAGE: magnetization prepared rapid gradient echo, MRI: magnetic resonance imaging, T1w: T1-weighted.

Only median intensities, extracted from unscaled bias-corrected T1w images, in the temporal fatty tissue (CoV = 0.139) and temporal muscle (CoV = 0.139) were in the range of cortical GM (CoV = 0.139) and WM (CoV = 0.130). All other candidate reference regions yielded CoVs ranging between 0.19 and 0.45. Consequently, we only implemented linear scaling with the median intensities of 1) fatty tissue and 2) temporal muscle as scaling factors. Regarding MPRAGE signal modeling, fixed T1 values for fatty tissue (T1 = 378.62 ms) and temporal muscle (T1 = 1491.44 ms) were calculated using all R1 maps (*n* = 1078). Additionally, we included the eye’s vitreous body (T1 = 4838.19 ms), which was the only accurately segmented region in the low T1w intensity range with reasonably low absolute T1w intensity variability (standard deviation: ±425.89 [a.u.]) compared to GM and WM (standard deviation: ± 1893.65 [a.u.] and ± 3191.54 [a.u.]), providing a stable reference point for orthogonal distance regression. Fig. 2 shows a representative illustration of the fitted MPRAGE signal modeling curve.

### 3.2. Variance analysis

**Fig. 3.**
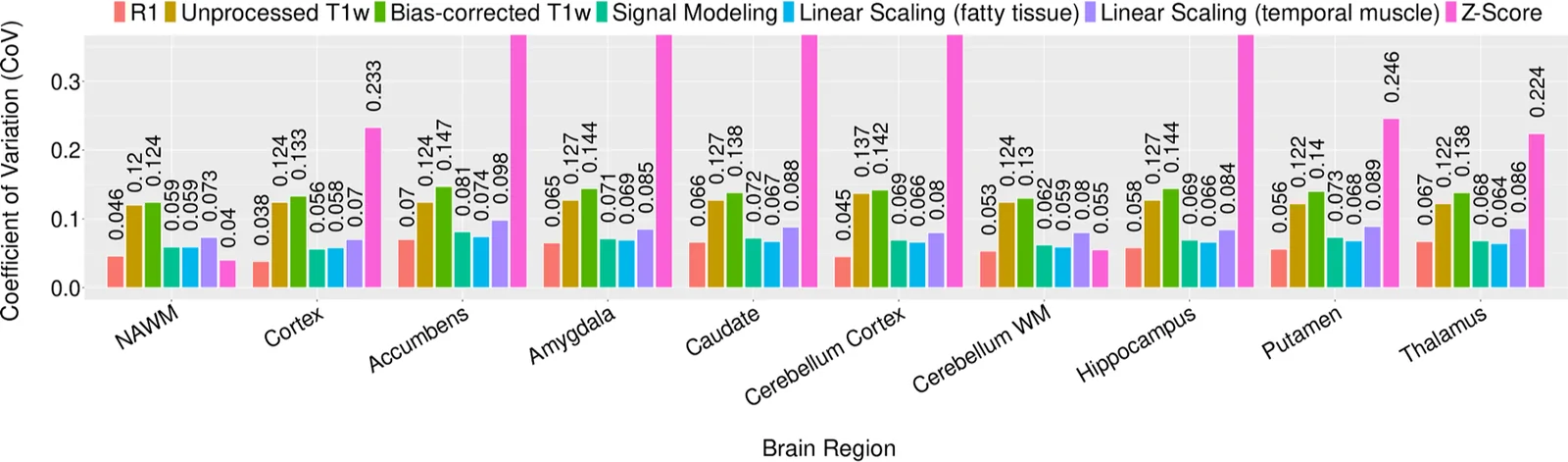
Inter-image variance in brain regions. The inter-image intensity variance is presented for each brain region and image type in terms of CoV. Linear scaling with fatty tissue and signal modeling consistently yields CoVs similar to R1 maps. Linear scaling with temporal muscle results in slightly larger CoVs; Z-score standardization provides the lowest CoVs in WM but the largest CoVs in all other regions (CoVs in the Accumbens, Amygdala, Caudate, Cerebellum Cortex, and Hippocampus are not completely displayed, and range between CoV = 0.6 and CoV = 3.2). Abbreviations: CoV: coefficient of variation, NAWM: normal-appearing white matter, T1w: T1-weighted, WM: white matter.

Signal modeling and linear scaling with fatty tissue consistently resulted in CoVs closest to those of R1 maps. Z-score standardization resulted in the lowest CoVs in NAWM and cerebellum WM but produced outliers (highest CoVs) in all other regions. Apart from these outliers, unprocessed and bias-corrected T1w images showed the largest CoVs in all regions. The CoVs per ROI of all image types are presented in Fig. 3.

### 3.3. Correlations with R1

**Fig. 4.**
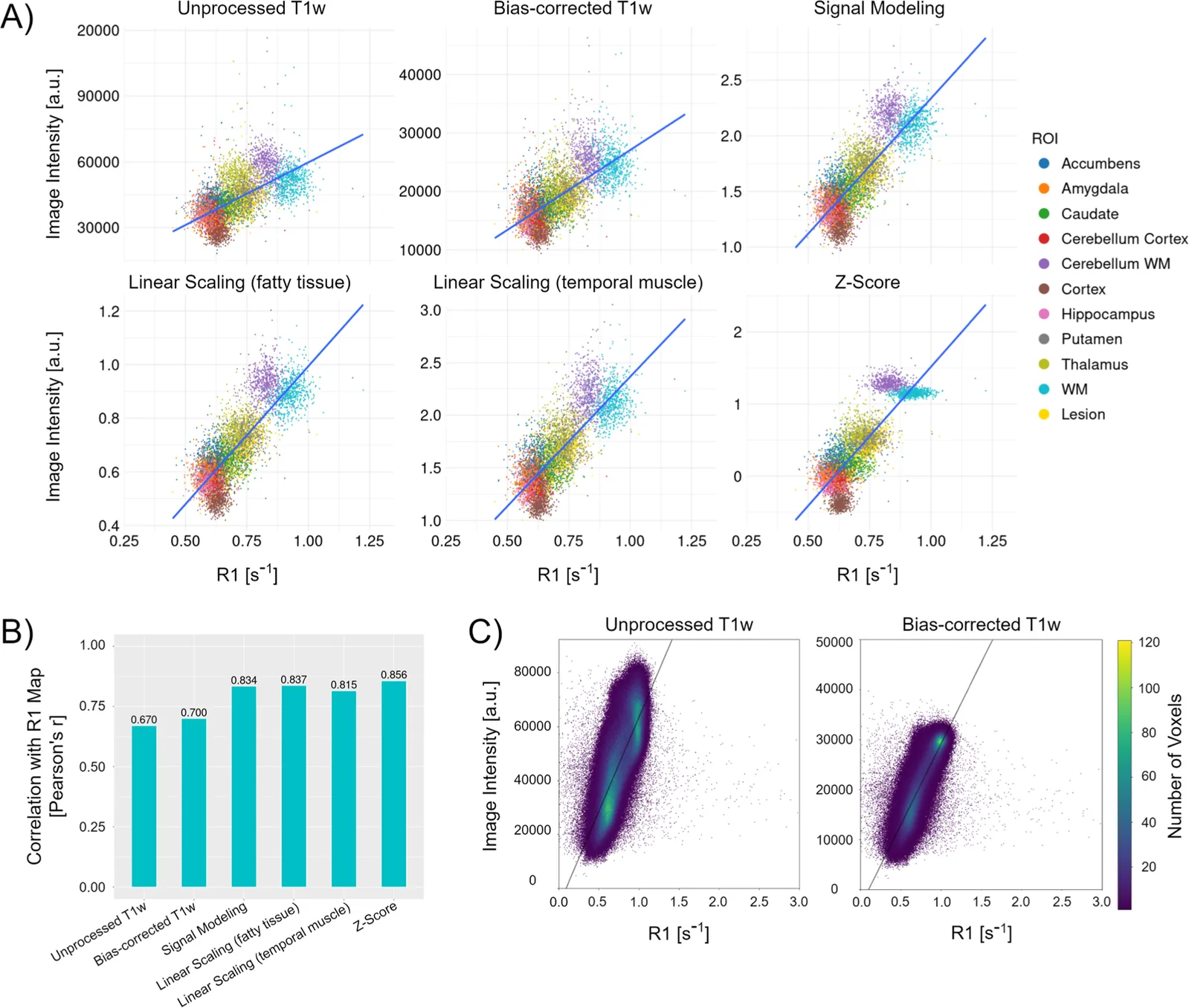
Correlation of scaled T1w image intensities with R1 values. The graphs showcase the correlation of the differently scaled T1w images with R1 maps. A) Correlation plots across brain regions. Each dot represents the median R1 value (x-axis) and the median intensity according to the selected image type (y-axis) within a brain region; brain regions are highlighted in different colors. The blue linear regression line shows the relation between the corresponding scaled T1w image intensities and R1 values. B) Pearson’s r values associated with the linear regressions from A). The strongest correlation with R1 was observed for z-score standardization (*r* = 0.86), followed by linear scaling with fatty tissue (*r* = 0.84) and MRI signal modeling (*r* = 0.83). C) Example of voxel-wise correlation. The voxel-wise correlation (Pearson’s r) with R1 is calculated per image and averaged across all images. The mean correlation with R1 was the same for each image type derived from bias-corrected T1w images (*r* = 0.84 ± 0.04) and was higher than that of unprocessed T1w images (*r* = 0.76 ± 0.05). The correlation plot of the unprocessed T1w image shows a much larger spread (y-axis) than that of the bias-corrected T1w image. The correlation plot was virtually the same for all other intensity scaling methods, apart from different scales of the y-axis, since the voxel-wise correlation within an image is invariant under linear transformation (i.e., intensity scaling). Abbreviations: ROI: region of interest, T1w: T1-weighted, WM: white matter.

Correlations between scaled T1w-based images and R1 maps are provided based on median intensities from multiple ROIs and for average voxel-wise correlations (see Fig. 4).

Across multiple brain regions, the highest correlation between scaled T1w images and R1 maps was observed for z-score standardization (*r* = 0.86), followed by linear scaling with fatty tissue (*r* = 0.84) and MPRAGE signal modeling (*r* = 0.83). The correlation plots of the different methods are shown in Fig. 4A, and Pearson’s r correlation values of all methods are shown in Fig. 4B

The averages of voxel-wise correlation values were similar for all image types (*r* = 0.84 ± 0.04) except for unprocessed T1w images (*r* = 0.76 ± 0.05). As expected, linear scaling and z-score standardization preserved the linear relationship of unscaled bias-corrected T1w image intensities with R1 values. This is also approximately true for signal modeling, as the conversion of T1w image intensities into estimated R1 (= 1/T1) values is a near-to-linear transformation being the inversion of the hyperbolic relation between T1 values and T1w signal intensities illustrated in Fig. 2. Exemplary voxel-wise correlation plots of T1w image intensities with R1 maps are shown in Fig. 4C.

### 3.4. Group comparisons based on EDSS

#### 3.4.1. Scaling factors

Only the scaling factor derived from z-score standardization (this method relies on the standard deviation of whole-brain intensities) differed significantly between high EDSS and low EDSS. Consequently, a group comparison of NAWM intensities was not conducted with z-score standardized T1w images.

#### 3.4.2. NAWM intensity

**Fig. 5.**
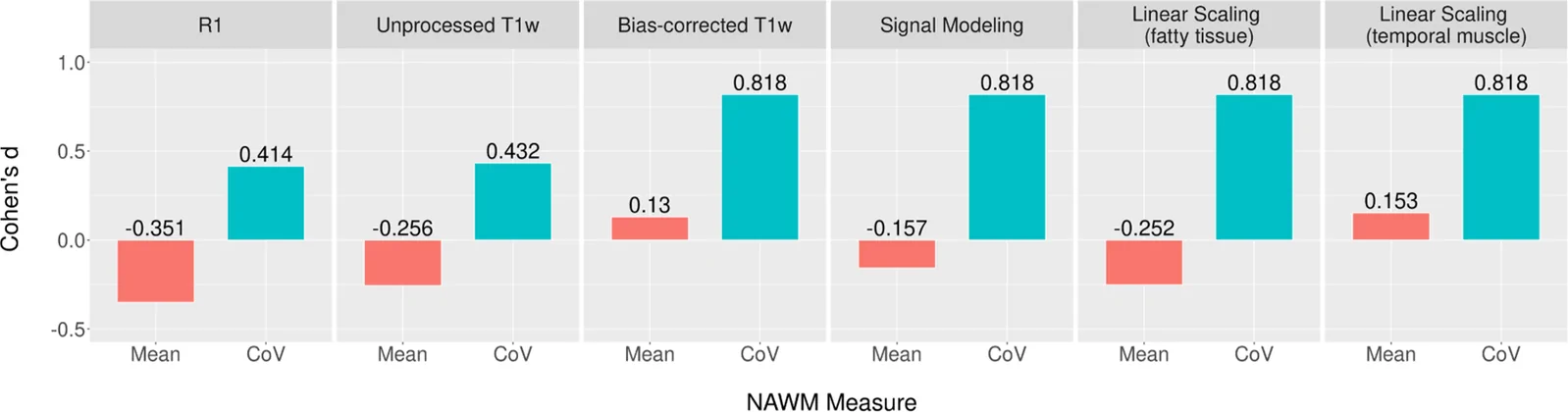
Group comparison effect size. The effect size (Cohen’s d) is presented for group comparison of mean and CoV of NAWM R1 and image intensities between low and high EDSS groups. Differences between low EDSS and high EDSS are more pronounced for CoVs than mean values and in opposite directions. Noticeably, CoVs extracted from bias-corrected T1w images yield a larger effect (*d* = 0.818) than measures extracted from R1 maps (Mean: *d* = −0.351, CoV: *d* = 0.414). Besides R1 maps, only unprocessed T1w images and linear scaling with fatty tissue show a small effect size regarding mean NAWM image intensity. Note that the difference in mean NAWM intensities was not statistically significant for unprocessed T1w images (see Table 3). All other image types exhibited negligible effect sizes with regard to mean NAWM intensity. Abbreviations: CoV: coefficient of variation, EDSS: expanded disability status scale, NAWM: normal-appearing white matter, T1w: T1-weighted.

As expected, mean R1 values in NAWM demonstrated a significant difference between low and high EDSS. Besides R1 maps, only linear scaling with fatty tissue revealed a significant group difference in mean NAWM intensities (see Table 3); of note, the effect size was similarly high (see Fig. 5).

Considering NAWM CoVs, significant group differences were observed across all image types. The most significant group differences were demonstrated by merely bias-corrected T1w images. Since CoVs are invariant under any linear scaling transformation, both linear scaling methods yielded the same results, while signal modeling yielded a very similar result. When age, sex, disease duration, and lesion volume were added as covariates, the differences only remained significant for bias-corrected T1w images. Detailed results are presented in Table 3. Additionally, Fig. 5 shows that effect sizes of group differences are much larger for CoVs than for mean intensities. Of note, the effect size of CoVs derived from bias-corrected T1w images (Cohen’s *d* = 0.818) is greater than any effect size from R1 maps (Cohen’s d of mean: −0.351, Cohen’s d of CoV: 0.414).

## 4. Discussion

This study compared and validated methods for converting conventional T1w MRI image intensities into (semi-) quantitative values. Besides quantitative R1 maps, the scaled T1w images were also compared to unprocessed T1w and bias-corrected T1w images to demonstrate the advantage over unscaled image intensities. To prevent the interference of brain pathology, such as in MS, we focussed on scaling methods avoiding brain regions as reference regions, which has only been attempted in a few studies (Brown et al., 2020; Ganzetti et al., 2014; Ghassemi et al., 2015; Lee et al., 2015; Wahid et al., 2021; Wiltgen et al., 2024b). We found that linear scaling with temporal fatty tissue yielded the best results concerning variance reduction, correlation with R1 maps, and group comparison (mild vs. severe MS-related disability) of NAWM intensity. Similarly, Ghassemi et al. (Ghassemi et al., 2015) found that a scaling method including fatty tissue yielded the best variance reduction and image harmonization results compared to unprocessed T1w images and Nyul’s method (Nyúl and Udupa, 1999).

We tested eight candidate regions with a data-driven approach to identify suitable reference regions outside the brain. The assumption was that the intensities of MRI images are affected by biological and technical factors and that a single linear factor can account for the latter (Brown et al., 2020; Haase, 1990). The biological inter-image variance in healthy brain WM and GM is generally low; however, both can be influenced by tissue damage in diseases affecting the brain. Nonetheless, this biological inter-image variance represents only a small fraction of the total inter-image variance compared to the technical inter-image variance. Therefore, intensities of regions outside the brain, suitable for estimating the scaling factor, should not exhibit a variance considerably higher than that of brain WM and GM. Only temporal fatty tissue and temporal muscle fulfilled this criterion. For the MRI signal modeling method, eyes were also selected due to their low standard deviation, making them suitable for orthogonal distance regression. To our knowledge, previous studies have not applied this approach; however, we consider the systematic identification of reference regions a crucial step to ensure reliable implementation of intensity scaling.

Intensity scaling methods can only be applied reliably if validated technically, serving as proof-of-concept, and biologically, confirming the appropriateness of scaling methods in the specific use case (in our case, MS). R1 maps served as a reference for validation since they are known to be sensitive to pathology in MS (Ladopoulos et al., 2022; Lommers et al., 2019; MacKay et al., 2009; Manfredonia et al., 2007; O’Muircheartaigh et al., 2019; Pontillo et al., 2023; Schmierer et al., 2004; Vrenken et al., 2006).

As part of the technical validation, reducing inter-image variance is a prerequisite for intensity scaling. However, it should not be the sole quality criterion, as minimizing variance can also lead to removing biological variance. For instance, Brown et al. showed that scaling methods with the highest inter-image variance reduction can remove biologically meaningful variance (Brown et al., 2020). The current study shows that linear scaling with fatty tissue and signal modeling yielded CoVs similar to those of R1 maps, achieving the most effective variance reduction across all brain ROIs (Fig. 3). Their slightly increased CoVs in comparison to R1 maps in bigger regions (e.g., NAWM and cortex) may be caused by less effective bias field correction in comparison to the measurement-based correction of the low-frequency bias field in MPM. Regarding the correlation with R1 maps, we found that bias correction expectedly increases the voxel-wise correlation compared to unprocessed T1w images (Fig. 4C), well compatible with the notion that bias correction decreases technical variance in the individual image while ignoring the absolute intensity scale. Interestingly, ROI-based correlations of scaled T1w images with R1 maps (Fig. 4A and Fig. 4B) were virtually the same for all scaling methods (R^2^ = 0.66–0.73), not allowing for a differentiated quality assessment. Brown et al. found similar correlation values (R^2^ = 0.63) between fat-scaled T1w images and MTR, a measurement that is sensitive to similar properties as R1 maps (e.g., density of macromolecules) (Brown et al., 2020). Our results indicate that scaling with regions outside the brain can yield correlations with R1 maps comparable to scaling methods based on WM, GM, and CSF, as (Hasse et al., 2022) reported in healthy subjects (R^2^ = 0.72).

Z-score standardization yielded seemingly promising results regarding the correlation with R1 maps and variance reduction in WM, but it also resulted in the largest CoVs in all other regions. In addition, the standard deviation used as a scaling factor in z-score standardization showed a significant difference between the two MS groups (mild and severe disability), indicating that the efficient reduction of technical variance goes along with removing biological variance. This is conceivable since the standard deviation is calculated across the entire brain and is, therefore, influenced by WM lesion load, GM atrophy, and NAWM damage. Hence, z-score standardization is not a reliable approach for intensity conversion of T1w images in the specific case of MS.

Regarding biological validation, results in Table 3 show that mean R1 values in NAWM significantly differed between groups (mild vs. severe disability), well in accordance with the literature, where a decrease in R1 values in NAWM has been associated with MS pathology (Ladopoulos et al., 2022; Lommers et al., 2019; MacKay et al., 2009; Manfredonia et al., 2007; O’Muircheartaigh et al., 2019; Pontillo et al., 2023; Schmierer et al., 2004; Vrenken et al., 2006). Notably, this group difference could also be demonstrated with scaled T1w images using fatty tissue, showing sensitivity with regard to MS-related disability.

In contrast to the mean NAWM intensity, a rather global tissue integrity measure, the NAWM CoV could reflect local alterations within one subject. The CoV is a scale-independent measure that can be directly derived from bias-corrected T1w images. To our surprise, the group difference between people with low (≤3) and high (>3) EDSS in NAWM was most significant when using the CoV instead of the mean values for all image types. Even after including age, sex, disease duration, and lesion volume as covariates, the CoV from bias-corrected T1w images yielded a significant difference. This was not the case in R1 maps since, in contrast to conventional T1w images, R1 maps are optimized for generating reliable quantitative intensities, which comes at the expense of a lower image resolution and tissue contrast. Similar results were found by Johnson et al. (Johnson et al., 2023), with CoVs of the MWF in NAWM differentiating more accurately between healthy controls and MS than the mean. Taken together, CoV and mean values within NAWM could provide complementary information on tissue integrity. On the one hand, CoV could be used to indicate disease-related widespread inhomogeneous tissue alterations. On the other hand, intensity scaling allows for calculating mean intensity values that could help quantify the global level of tissue damage (e.g., demyelination in MS). Overall, group differences in mean and CoVs of NAWM from scaled T1w images show the potential for retrospective intensity-based analyses of conventional T1w images to identify new imaging biomarkers associated with the progression of diseases such as MS.

We acknowledge the limitations of our study. The MRI images were acquired on the same scanner and with the same protocol. T1w sequences are optimized for brain tissues; coverage of regions outside the brain may vary between scanners and protocols, and so may their suitability as reference regions. Finally, our study was limited to brain MRI of people with MS. Consequently, we regard a validation of intensity scaling based on temporal fatty tissue in different T1w sequences in a multi-center study, ideally across multiple neuropsychiatric diseases, as a reasonable next step. In the same context, the advantage of using a single reference region (temporal fatty tissue) over multiple reference regions (signal modeling) needs to be confirmed.

## 5. Conclusion

Temporal fatty tissue is a promising candidate for a valuable reference region to convert conventional T1w image intensities into (semi-) quantitative values with high inter-image comparability when quantitative T1 maps are not available. In addition to absolute intensity measures, we found that changes in MS NAWM appear to increase intensity variability detectable in conventional T1w images. This observation is noteworthy and warrants further investigation.

## Supplementary Material

1

Supplementary material associated with this article can be found, in the online version, at doi:10.1016/j.neuroimage.2025.121395.

## Figures and Tables

**Table 1:** Cohort characteristics.

		Number of subjects	Number of imaging data sets	Female/Male	Diagnosis	Age in years mean ± sd	Lesion volume in mm3 median (IQR)	EDSS median (IQR)	Disease duration
**Total cohort**		701	1078	682 / 396	CIS: 39 RRMS: 960 SPMS: 22 PPMS: 40 RIS: 17	39.90 ± 11.03	2543.7 (859.8 – 7098.78)	1.5 (0.0 – 2.0)	6.7 (2.5 – 11.5)
**Validation cohort**	**Total**	597	597	376 / 221	CIS: 22 RRMS: 541 SPMS: 14 PPMS: 20	40.48 ± 11.12	2667.1 (866.1 – 7121.7)	1.5 (0.0 – 2.0)	7.1 (3.1 – 11.5)
	**Low EDSS**	526	526	330 / 196	CIS: 22 RRMS: 499 SPMS: 0 PPMS: 5	39.47 ± 10.61	2309.3 (781.2 – 5995.1)	1.0 (0.0 – 2.0)	6.7 (2.9 – 11.0)
	**High EDSS**	71	71	46 / 25	CIS: 0 RRMS: 42 SPMS: 14 PPMS: 15	47.94 ± 12.04	7752.8 (3499.9 – 18,147.6)	4.5 (3.5 – 6.0)	11.8 (5.3 – 21.7)

Abbreviations: CIS: clinically isolated syndrome, EDSS: expanded disability status scale, IQR: interquartile range, MS: multiple sclerosis, PPMS: primary progressive multiple sclerosis, RRMS: relapsing-remitting multiple sclerosis, RIS: radiologically isolated syndrome, sd: standard deviation, SPMS: secondary progressive multiple sclerosis.

**Table 2:** MRI acquisition parameters.

	3D T1w (MPRAGE)	3D FLAIR	Multiparameter mapping			
			T1w	PDw	MTw	B1 map
Reconstruction voxel size	0.75×0.75×0.75 mm^3^	0.75×0.75×0.75 mm^3^	1 × 1 × 1 mm^3^	1 × 1 × 1 mm^3^	1 × 1 × 1 mm^3^	1 × 1 × 2.5 mm^3^
Acquisition voxel size	1 × 1 × 1 mm^3^	1 × 1 × 1 mm^3^	1 × 1 × 1 mm^3^	1 × 1 × 1 mm^3^	1 × 1 × 1 mm^3^	3.5 × 3.5 × 5 mm^3^
TR	9 ms	4800 ms	18 ms	18 ms	48 ms	30/150 ms
TE	4 ms	shortest	6 echoes first: 2.4 ms echo spacing: 2.4 ms	6 echoes first: 2.4 ms echo spacing: 2.4 ms	6 echoes first: 2.4 ms echo spacing: 2.4 ms	shortest
TI	1000ms	1650 ms	/	/	/	/
Flip angle	8°	90°	25°	4°	6°	60°
Acquisition mode	turbo field echo	turbo spin echo	fast field echo	fast field echo	fast field echo	fast field echo

Abbreviations: FLAIR: fluid-attenuated inversion recovery, MPRAGE: magnetization-prepared rapid gradient echo, MTw: magnetization transfer-weighted, PDw: proton density-weighted, T1w: T1-weighted, TE: echo time, TI: inversion time, TR: repetition time.

**Table 3:** NAWM group comparison.

	NAWM Mean intensity			NAWM CoV		
	Low EDSS	High EDSS	p-value	Low EDSS	High EDSS	p-value
**R1**	0.972 ± 0.044	0.956 ± 0.047	**0.0081**	0.070 ± 0.013	0.076 ± 0.015	**0.0027**
**Unprocessed T1w**	53,873.9 ± 6154.9	52,025.8 ± 8156.6	0.0695	0.109 ± 0.008	0.114 ± 0.012	**0.0031**
**Bias-corrected T1w**	25,187.8 ± 3084.7	25,610.6 ± 3391.1	0.3218	0.062 ± 0.010	0.072 ± 0.015	**1.636E-07** *
**Signal modeling**	2.155 ± 0.128	2.136 ± 0.107	0.1833	0.062 ± 0.010	0.073 ± 0.015	**1.648E-07** *
**Linear scaling (fatty tissue)**	0.918 ± 0.055	0.905 ± 0.046	**0.0344**	0.062 ± 0.010	0.072 ± 0.015	**1.636E-07** *
**Linear scaling (temporal muscle)**	2.174 ± 0.160	2.197 ± 0.137	0.1989	0.062 ± 0.010	0.072 ± 0.015	**1.636E-07** *

Bold numbers indicate p-values < 0.05, i.e., statistically significant results.

Abbreviations: CoV: coefficient of variation, EDSS: expanded disability status scale, NAWM: normal-appearing white matter, T1w: T1-weighted.

* remains significant in ANCOVA with age, sex, disease duration, and lesion volume as covariates.

## Data Availability

Data used and generated during this study are private and not publicly available, but could be made available upon reasonable request.

## Glossary

CIS: clinically isolated syndrome
CoV: coefficient of variation
EDSS: expanded disability status scale
FLAIR: fluid-attenuated inversion recovery
GM: gray matter
LST-AI: lesion segmentation tool – artificial intelligence
MNI: Montreal Neurological Institute
MPM: multiparameter mapping
MPRAGE: magnetization-prepared rapid gradient echo
MRI: magnetic resonance imaging
MS: multiple sclerosis
MTw: magnetization transfer-weighted
MWF: myelin water fraction
NAWM: normal-appearing white matter
PDw: proton density-weighted
ROI: region of interest
RIS: radiologically isolated syndrome
SAMSEG: sequence adaptive multimodal segmentation
T1w: T1-weighted
T2w: T2-weighted
TE: echo time
TI: inversion time
TR: repetition time
WM: white matter
