# Supplementary material for: Towards quantitative intensity analysis of conventional T1-weighted images in multiple sclerosis
Source: Neuroimage. Author manuscript; Available in PMC 2026 Aug 28. (PMC13523021; doi:10.1016/j.neuroimage.2025.121395)
Supplement: Supplementary file 1 [file NIHMS2180171-supplement-1.docx]

SUPPLEMENTARY MATERIAL

1. MPRAGE signal equation

The MPRAGE signal equation is derived based on information from literature (Brant-Zawadzki et al., 1992; Deichmann et al., 2000; Deichmann and Haase, 1992; Haase, 1990; Vernier et al., 2021). A sequence diagram of the applied T1w MPRAGE sequence is presented in Figure 1.

Figure 1 MPRAGE sequence diagram

The MPRAGE sequence starts with the image acquisition once the steady-state equilibrium magnetization M^eq^ is reached. One block begins with a 180° inversion pulse, followed by an inversion time TI. Next, the RAGE block, consisting of N_total_ phase encoding steps, starts. Each phase encoding step includes the application of an RF pulse with flip angle $\alpha$, and a gradient readout phase after the echo time TE has passed. Two consecutive RF pulses are separated by the repetition time TR. After the RAGE block, the steady-state equilibrium magnetization is reached again after the recovery time T_rec_. Then, the next MPRAGE block starts with applying the next inversion pulse. Abbreviations: MPRAGE: magnetization-prepared rapid gradient echo, RAGE: rapid gradient echo, RF: radio frequency, TE: echo time, TI: inversion time, TR: repetition time, T_rec_: recovery time.

In general, the longitudinal magnetization recovers over time as follows:

$$M\left( t \right)=M\left( 0 \right)*e^{-\frac{t}{T1}}+M_{0}(1-e^{-\frac{t}{T1}})$$

Here, $M_{0}$ represents the general acquisition-specific equilibrium magnetization. The longitudinal magnetization after an excitation pulse with flip angle alpha is:

$$M=M\left( 0 \right)*cos(\alpha)$$

Note that the MPRAGE image acquisition starts when the steady state $M_{i}^{eq}$ is reached, which also implies the following condition: $M_{i}^{eq}= M_{i-1}^{eq}$.

With these two formulas, we can derive the equation of the MPRAGE signal. One MPRAGE block consists of an inversion time TI until the rapid gradient echo (RAGE) block starts, one RAGE block, and a recovery time after the RAGE block until the next inversion pulse. The RAGE block consists of N phase encoding steps, each one includes a low-angle RF pulse and a gradient echo readout. In the following we use:

$$E_{TR}=e^{-\frac{TR}{T1}}, E_{TI}=e^{-\frac{TI}{T1}}, E_{rec}=e^{-\frac{T_{rec}}{T1}}$$

1) The longitudinal magnetization after TI after the 180° inversion pulse is given by:

$$M_{i}^{0}=-M_{i-1}^{eq}*E_{TI}+M_{0}\left( 1-E_{TI} \right), with \cos\left( 180^{\circ} \right)=-1$$

2) After the first phase encoding step, i.e. the application of a low-angle RF pulse and magnetization recovery time TR (time between two low-angle RF pulses), the longitudinal magnetization is described by:

$$M_{i}^{1}=M_{i}^{0}*cos(\alpha)*E_{TR}+M_{0}(1-E_{TR})$$

Here, cos(α) relates to the effect of the small angle RF pulse on the longitudinal magnetization and the rest of the terms describe the longitudinal magnetization recovery over time.

3) After the second phase encoding step, the longitudinal magnetization is described by:

$$M_{i}^{2}=M_{i}^{1}*\cos\left( \alpha\right)*E_{TR}+M_{0}\left( 1-E_{TR} \right)$$

$$=\left[ M_{i}^{0}*\cos\left( \alpha\right)*E_{TR}+M_{0}\left( 1-E_{TR} \right) \right]*\cos\left( \alpha\right)*E_{TR}+M_{0}\left( 1-E_{TR} \right)$$

$$=M_{i}^{0}*\left( \cos\left( \alpha\right)*E_{TR} \right)^{2}+M_{0}\left( 1-E_{TR} \right)*(\cos\left( \alpha\right)*E_{TR}+1)$$

4) After the third phase encoding step, the longitudinal magnetization is described by:

$$M_{i}^{3}=M_{i}^{2}*\cos\left( \alpha\right)*E_{TR}+M_{0}\left( 1-E_{TR} \right)$$

$$=\left[ M_{i}^{0}*\left( \cos\left( \alpha\right)*E_{TR} \right)^{2}+M_{0}\left( 1-E_{TR} \right)*(\cos\left( \alpha\right)*E_{TR}+1) \right]*\cos\left( \alpha\right)*E_{TR}+M_{0}\left( 1-E_{TR} \right)$$

$$=M_{i}^{0}*\left( \cos\left( \alpha\right)*E_{TR} \right)^{3}+M_{0}\left( 1-E_{TR} \right)*[\left( \cos\left( \alpha\right)*E_{TR} \right)^{2}+cos \left( \alpha\right)*E_{TR}+1)]$$

5) If we continue iterating, we find the following expression for the N-th phase encoding step:

$$M_{i}^{N}=M_{i}^{0}*\left( \cos\left( \alpha\right)*E_{TR} \right)^{N}+M_{0}\left( 1-E_{TR} \right)*\sum_{n=0}^{N-1} \left( \cos\left( \alpha\right)*E_{TR} \right)^{n}$$

6) With the geometric series:

$$M_{i}^{N}=M_{i}^{0}*\left( \cos\left( \alpha\right)*E_{TR} \right)^{N}+M_{0}\left( 1-E_{TR} \right)*\frac{{1-\left( \cos\left( \alpha\right)*E_{TR} \right)}^{N}}{1-\cos\left( \alpha\right)*E_{TR}}$$

7) Including the expression of $M_{i}^{0}$ from step 1):

$$M_{i}^{N}$$

$${=M}_{0}\left( 1-E_{TR} \right)*\frac{{1-\left( \cos\left( \alpha\right)*E_{TR} \right)}^{N}}{1-\cos\left( \alpha\right)*E_{TR}}+(\left( -M_{i-1}^{eq} \right)*E_{TI}+M_{0}\left( 1-E_{TI} \right))*\left( \cos\left( \alpha\right)*E_{TR} \right)^{N}$$

$$=M_{0}\left( 1-E_{TR} \right)*\frac{{1-\left( \cos\left( \alpha\right)*E_{TR} \right)}^{N}}{1-\cos\left( \alpha\right)*E_{TR}}-M_{i-1}^{eq}*E_{TI}*\left( \cos\left( \alpha\right)*E_{TR} \right)^{N}+M_{0}(1-E_{TI}))*\left( \cos\left( \alpha\right)*E_{TR} \right)^{N}$$

8) Considering the recovery time after the RAGE block, we get for the steady state equilibrium magnetization (which is different from $M_{0}$) at the end of the MPRAGE block:

$$M_{i}^{eq}$$

$$=M_{0}\left( 1-E_{TR} \right)*\frac{{1-\left( \cos\left( \alpha\right)*E_{TR} \right)}^{N_{total}}}{1-\cos\left( \alpha\right)*E_{TR}}*E_{rec}-M_{i-1}^{eq}*E_{TI}*\left( \cos\left( \alpha\right)*E_{TR} \right)^{N_{total}}*E_{rec}$$

$$+ M_{0}(1-E_{TI}))*\left( \cos\left( \alpha\right)*E_{TR} \right)^{N_{total}}*E_{rec}+M_{0}(1-E_{rec})$$

Notice how the total number of phase encoding steps in one RAGE block N=N_total_ is used to calculate the steady state equilibrium magnetization.

9) In the steady state, $M_{i}^{eq}=M_{i-1}^{eq}$ must hold, which leads to the following expression:

$$M^{eq}$$

$$=M_{0}*\frac{1-E_{rec}+\left( 1-E_{TR} \right)*\frac{{1-\left( \cos\left( \alpha\right)*E_{TR} \right)}^{N_{total}}}{1-\cos\left( \alpha\right)*E_{TR}}*E_{rec}+(1-E_{TI})*\left( \cos\left( \alpha\right)*E_{TR} \right)^{N_{total}}*E_{rec}}{1+\left( \cos\left( \alpha\right)*E_{TR} \right)^{N_{total}}*E_{TI}*E_{rec}}$$

If we want to model the signal after the i-th readout pulse $S_{i}=\sin\alpha*M_{i}^{N}*e^{-\frac{TE}{{T2}^{*}}}$, we need to consider at which step of the RAGE block the center of the k-space was sampled because the center of the k-space encodes the contrast. Therefore, if the center is sampled in the n-th step, then the magnetization of this step should be used (i.e., select N=n). Our MPRAGE uses linear cartesian profile order for k-space filling. Hence, we should use N=N_total_/2.

Parameters of the in-house MPRAGE sequence:

- Shot interval = 3000ms
- TI/prepulse delay time = 1000ms
- TE = 4ms
- TR = 9ms
- FA = 8°
- N_total_ = 48 (TFE shots)
- T_rec_ = shot interval – TI – N_total_ * TR = 3000-1000-48*9 = 1568ms

2. Atlas generation

The anatomical atlas for the extra-cerebral tissues was constructed from an existing data set of manual segmentations (Farcito et al., 2019), where subcutaneous fat was included as an additional tissue. The fat segmentations were done manually on mDixon scans included in the data set, which have excellent fat contrast. The anatomical atlas was then re-built from these segmentations using the approach described in a previous study (Puonti et al., 2020). The new anatomical atlas improves the segmentation accuracy of the non-brain structures compared to the original atlas, which did not explicitly model the subcutaneous fat (Diedrichsen et al., 2025).

3. Segmentation of temporal fatty tissue and temporal muscle

The manual segmentation of temporal fatty tissue and temporal muscle was conducted on T1w images. Regarding the temporal fatty tissue, the entire connected hyperintense structure of the temporal fatty tissue was segmented to include enough voxels that enable the reliable calculation of mean intensities. Regarding the temporal muscle, the connected structure was segmented starting at a lower limit defined by the slice on which the temporal muscle approximately extends from the eyes to the ears and ending at an upper limit defined by the slice on which the frontal horn of the lateral ventricles appears. Despite inter-subject variations of these upper and lower limits, the temporal muscle region is large and includes a great number of voxels, independent of these variations, and enables the reliable calculation of mean intensities. The manual segmentation was conducted by a neuroscientist with 4 years of experience with brain MRI segmentation and was supervised by an experienced neurologist (15 years of experience).

4. Curve fitting results

The success of the fitting process was evaluated by calculating the R^2^ and Normalized Root Mean Squared Error (NRMSE) for each image and reporting the mean with the standard deviation and the median with the interquartile range (IQR). We found a mean R^2^ value of 0.91 +/- 0.04, and a median of 0.92 (0.90-0.94). The NRMSE values showed a mean of 0.12 +/- 0.03, and a median of 0.11 (0.10-0.13).

REFERENCES

Brant-Zawadzki, M., Gillan, G.D., Nitz, W.R., 1992. MP RAGE: a three-dimensional, T1-weighted, gradient-echo sequence--initial experience in the brain. Radiology 182, 769–775. https://doi.org/10.1148/radiology.182.3.1535892

Deichmann, R., Good, C.D., Josephs, O., Ashburner, J., Turner, R., 2000. Optimization of 3-D MP-RAGE Sequences for Structural Brain Imaging. NeuroImage 12, 112–127. https://doi.org/10.1006/nimg.2000.0601

Deichmann, R., Haase, A., 1992. Quantification of *T*1 values by SNAPSHOT-FLASH NMR imaging. Journal of Magnetic Resonance (1969) 96, 608–612. https://doi.org/10.1016/0022-2364(92)90347-A

Diedrichsen, M., Merhout, J., Nielsen, J. D, Greve, D. N., Thielscher, A. and Puonti, O., 2025. Modeling subcutaneous fat improves skull segmentation for individualized volume conductor head models. *Accepted in* *2025 47th Annual International Conference of the IEEE Engineering in Medicine and Biology Society (EMBC)*. IEEE.

Farcito, S., Puonti, O., Montanaro, H., Saturnino, G.B., Nielsen, J.D., Madsen, C.G., Siebner, H.R., Neufeld, E., Kuster, N., Lloyd, B.A., Thielscher, A., 2019. Accurate anatomical head segmentations: a data set for biomedical simulations, in: 2019 41st Annual International Conference of the IEEE Engineering in Medicine and Biology Society (EMBC). Presented at the 2019 41st Annual International Conference of the IEEE Engineering in Medicine and Biology Society (EMBC), pp. 6118–6123. https://doi.org/10.1109/EMBC.2019.8857041

Haase, A., 1990. Snapshot flash mri. applications to t1, t2, and chemical-shift imaging. Magnetic Resonance in Medicine 13, 77–89. https://doi.org/10.1002/mrm.1910130109

Puonti, O., Van Leemput, K., Saturnino, G.B., Siebner, H.R., Madsen, K.H., Thielscher, A., 2020. Accurate and robust whole-head segmentation from magnetic resonance images for individualized head modeling. NeuroImage 219, 117044. https://doi.org/10.1016/j.neuroimage.2020.117044

Vernier, B., Van Reeth, E., Pilleul, F., Beuf, O., Ratiney, H., 2021. MRI Contrast Enhancement of Magnetization Prepared Steady State Sequence: An Optimal Control Framework, in: 2021 IEEE 18th International Symposium on Biomedical Imaging (ISBI). Presented at the 2021 IEEE 18th International Symposium on Biomedical Imaging (ISBI), pp. 1709–1713. https://doi.org/10.1109/ISBI48211.2021.9434151
